# Comparative Transcriptome Analysis of Salt-Tolerant and -Sensitive Soybean Cultivars under Salt Stress

**DOI:** 10.3390/ijms25189818

**Published:** 2024-09-11

**Authors:** Ye Cheng, Xiangqiang Cheng, Kai Wei, Yan Wang

**Affiliations:** Xinjiang Key Laboratory of Biological Resources and Genetic Engineering, College of Life Science and Technology, Xinjiang University, Urumqi 830049, China; chengye1121@163.com (Y.C.); 107552100982@stu.xju.edu.cn (X.C.)

**Keywords:** soybean, RNA-seq, comparative transcriptomes, salt stress

## Abstract

Soil salinity is a major limiting factor in soybean (*Glycine max* (L.) Merr.) yield in Xinjiang, China. Therefore, breeding soybean to tolerate highly saline soils is crucial to improve its yield. To explore the molecular mechanisms underlying the response of soybean to salt stress, we performed a comparative transcriptome analysis of root and leaf samples collected from two local soybean cultivars. The salt-tolerant cultivar ‘Xin No. 9’ (X9) showed higher photosynthetic activity than the salt-sensitive cultivar ‘Xinzhen No. 9’ (Z9) under salt stress. In total, we identified 13,180 and 13,758 differential expression genes (DEGs) in X9 and Z9, respectively, of which the number of DEGs identified in roots was much higher than that in leaves. We constructed the co-expression gene modules and conducted Gene Ontology (GO) term and Kyoto Encyclopedia of Genes and Genomes (KEGG) pathway enrichment analyses. The results suggested there were distinct differences in the mechanisms of response to salt stress between the two soybean cultivars; i.e., the salt-tolerant cultivar X9 exhibited alterations in fundamental metabolism, whereas the salt-sensitive cultivar Z9 responded to salt stress mainly through the cell cycle. The possible crosstalk among phytohormone signaling, MAPK signaling, phenylpropanoid biosynthesis, starch and sucrose metabolism, and ribosome metabolism may play crucial roles in the response to salt stress in soybean. Our results offered a comprehensive understanding of the genes and pathways involved in the response to salt stress in soybean and provided valuable molecular resources for future functional studies and the breeding of soybean varieties with enhanced tolerance to salinity.

## 1. Introduction

Soybean (*Glycine max* (L.) Merr.), which originated in China and was domesticated approximately 6000–9000 years ago, is an enormous source of both protein and edible oil [1]. As one of the most important oilseed crops worldwide, soybean can provide up to 69% of the vegetable protein and 30% of the oil needed in the human diet [2]. Global soybean production is increasing substantially to ensure global food supplies for the rapidly growing population in the world. However, soybean yields are severely challenged by unfavorable environmental factors. Soil salinization is one of the major stresses in agriculture and can greatly limit plant growth and development, leading to reduced crop yields. Soybean has a short growth cycle and is usually categorized as a moderately salt-tolerant crop [3,4]. Soybean yield can be reduced by more than 40% under salt stress [5,6], and high salinity can even result in soybean plant death [4,7]. Therefore, understanding the molecular mechanism of salt tolerance and improving the salt tolerance of soybean are major breeding targets around the world.

When exposed to salt stress, soybeans undergo osmotic stress, ionic toxicity, and a range of complex secondary effects. A high concentration of sodium ions (Na^+^) in the soil results in an elevated osmotic pressure, which disrupts cellular ion homeostasis and inhibits the uptake of water and nutrients. Consequently, this adversely affects plant growth and diminishes agricultural yield [8,9,10]. Furthermore, salt stress is associated with the accumulation of reactive oxygen species (ROS), which serve as a secondary stressor by inducing the peroxidation of membrane lipids and leading to the degradation of cellular membrane structures and proteins [11,12]. Therefore, soybean has accordingly evolved multiple tolerance mechanisms to cope with these adverse physiological changes under salt stress. For example, several genes participate in maintaining ion homeostasis under salt stress, such as high-affinity K^+^ transporter 1 (GmHKT1) and salt overly sensitive 1 (GmSOS1) [13,14,15]. Moreover, plant hormones, such as auxins, gibberellins (GAs), cytokinins (CKs), and abscisic acid (ABA), also play major roles in mediating plant responses to salt stress [16,17]. A comparative transcriptome study also revealed ABA and auxin signaling pathways responded to salt stress in soybean [18]. Moreover, some fundamental processes of soybean’s metabolism and cell cycle also play crucial roles in its response to salt stress, including starch, sucrose, phenylpropanoid metabolism, and ribosome pathways [18,19,20,21]. These studies underscore the intricate regulatory mechanisms governing the various pathways involved in soybean’s response to salt stress. However, there remains a significant gap in our understanding of the salt signaling processes that facilitate soybean’s adaptation to elevated salinity levels. This deficiency in knowledge has considerably hindered efforts to improve salt tolerance in soybean.

The analysis of differential gene expression between contrasting genotypes is instrumental in identifying candidate genes and elucidating underlying molecular mechanisms. A comparative transcriptome analysis between the salt-tolerant soybean cultivar ‘Qi Huang No. 34’ (QH34) and salt-sensitive cultivar ‘Dong Nong No. 50’ (DN50) reveals gene regulatory networks under salt stress in soybean [18]. Xinjiang is one of the regions with the most serious soil salinization in China, and soil salinization is one of the main reasons for soil health and ecosystem deterioration in Xinjiang, which has the most degraded arid and semiarid areas [22,23]. For this reason, breeders have selected several soybean cultivars to adapt to high-salinity soils in Xinjiang.

In this study, we selected two local soybean cultivars from Xinjiang, China: a salt-tolerant soybean cultivar ‘Xin No. 9’ (X9), which is a high-oil inbred line with high yields, and the salt-sensitive soybean cultivar ‘Xinzhen No. 9’ (Z9). We used a comparative transcriptomics analysis to reveal important pathways and identify candidate genes that responded to salt stress in soybean. The identification of candidate genes and their associated mechanisms will establish a novel foundation for the breeding of salt-tolerant and high-yield soybean varieties, thereby contributing to the enhancement of soybean productivity.

## 2. Results

### 2.1. Phenotypic and Photosynthetic Response of Soybean Seedlings to Salt Stress

**Figure 1:**
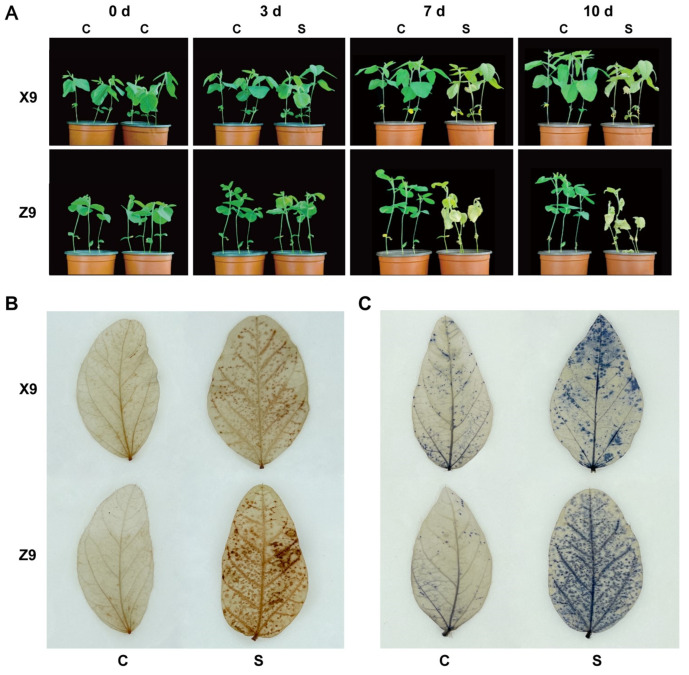
The phenotypic responses of two soybean cultivars to salt stress. (**A**) The phenotypic characteristics of soybean seedlings subjected to salt stress over varying durations. (**B**) The application of nitroblue tetrazolium (NBT) staining to soybean leaves exposed to salt stress. (**C**) The application of diaminobenzidine tetrahydrochloride (DAB) staining to soybean leaves exposed to salt stress. Abbreviations: C denotes the control condition with water; S denotes the condition of salt stress induced using 300 mM NaCl.

The two soybean cultivars studied in this study include a salt-tolerant cultivar, designated as X9, and a salt-sensitive cultivar, referred to as Z9. Both cultivars were treated with 300 mM NaCl solution for 10 days during the seedling stage. The results indicated that salt stress inhibited the growth of the soybean seedlings of both varieties. As the duration of exposure to salt stress increased, the leaves of both cultivars exhibited progressive curling and wilting (Figure 1A). Notably, the salt-sensitive cultivar Z9 demonstrated a more pronounced response to salt stress compared to the salt-tolerant cultivar X9 after seven days of treatment. Furthermore, staining with nitroblue tetrazolium (NBT) and diaminobenzidine tetrahydrochloride (DAB) showed that the leaves of the salt-sensitive Z9 cultivar exhibited darker staining than those of the salt-tolerant X9, suggesting that Z9 accumulated higher levels of ROS under salt stress (Figure 1B,C).

**Figure 2:**
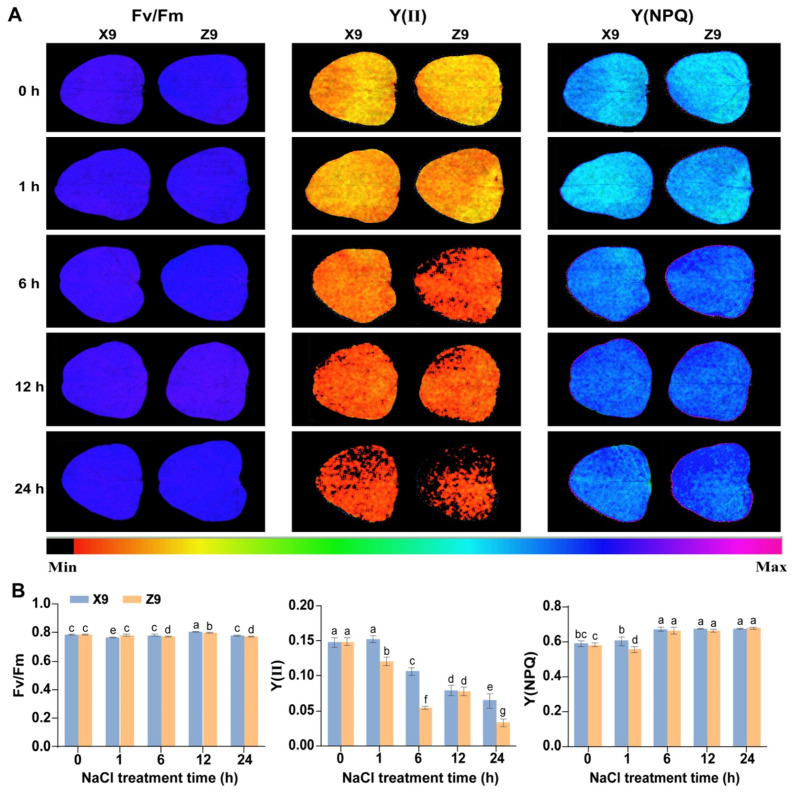
The photosynthetic response of two soybean cultivars to salt stress. (**A**) Chlorophyll fluorescence images of leaves. (**B**) The comparative analyses of three chlorophyll fluorescence parameters between two soybean cultivars. Each parameter in the histogram is represented as the mean and the error bars as the standard deviation (*n* = 3). Letters on the bars indicate significance of differences. Duncan’s test was used to calculate the significance of differences (*p* ≤ 0.05).

We subsequently tested the photosynthetic activity of soybean plants under salt stress by measuring three chlorophyll fluorescence parameters: Fv/Fm, Y(II), and Y(NPQ) at intervals of 0 h, 1 h, 6 h, 12 h, and 24 h (Figure 2A). The Fv/Fm fluorescence intensity remained constant for both soybean cultivars throughout the 24 h period of salt stress, with no significant differences observed between the two cultivars (Figure 2A,B). The Y(II) parameter exhibited a gradual decline over time in the X9 cultivar, while in the Z9 cultivar, Y(II) decreased from 0 to 6 h of salt stress, increased from 6 to 12 h, and subsequently decreased again, reaching a minimum at 24 h. Y(II) was significantly higher in the X9 cultivar compared to the Z9 cultivar (Duncan’s test, *p* ≤ 0.05), with the exception of measurements taken at 0 h and 12 h (Figure 2A,B). These findings suggested that the photosynthetic capacity of the X9 cultivar is stronger than that of the Z9 cultivar under salt stress. Y (NPQ) is an important indicator of photoprotection, demonstrated an increasing trend over time for both soybean cultivars following salt stress, indicating that both cultivars possess the ability to regulate light energy as a protective mechanism under such conditions. Overall, the leaves of both soybean cultivars exhibited a reduced chlorophyll content and inhibited photosynthesis in response to salt stress. However, the photosynthetic efficiency of the salt-tolerant X9 cultivar remained higher than that of the salt-sensitive Z9 cultivar. Consequently, we collected root and leaf samples at 0 h, 1 h, 6 h, 12 h, and 24 h post-salt treatment, as well as samples from the control plants, for RNA sequencing, ensuring three biological replicates.

### 2.2. Quality Control, Assembly, and Read Mapping of RNA-Seq Data

To achieve a comprehensive understanding of the gene expression profile of soybean plants subjected to salt stress, total RNA sequencing was conducted on the roots and leaves of two soybean cultivars following their exposure to salt stress. Each tissue and treatment condition was replicated three times, resulting in a total of 24 transcriptome datasets. After performing quality control procedures on the raw data, a total of 148.35 Gb of clean data were obtained, with each sample exceeding 5.77 Gb. The Q30 values for the 24 libraries exceeded 93.45%, while the GC content ranged from 43.26% to 44.34% (Table S1). Clean reads for each sample were aligned to the reference genome, achieving mapping efficiencies between 95.38% and 97.28% (Table S1). In total, 48,732 genes were identified as expressed across the 24 samples. Specifically, 47,193 genes were expressed in X9, and 46,786 genes were expressed in Z9, with 1946 genes uniquely expressed in X9 and 1539 genes uniquely expressed in Z9.

**Figure 3:**
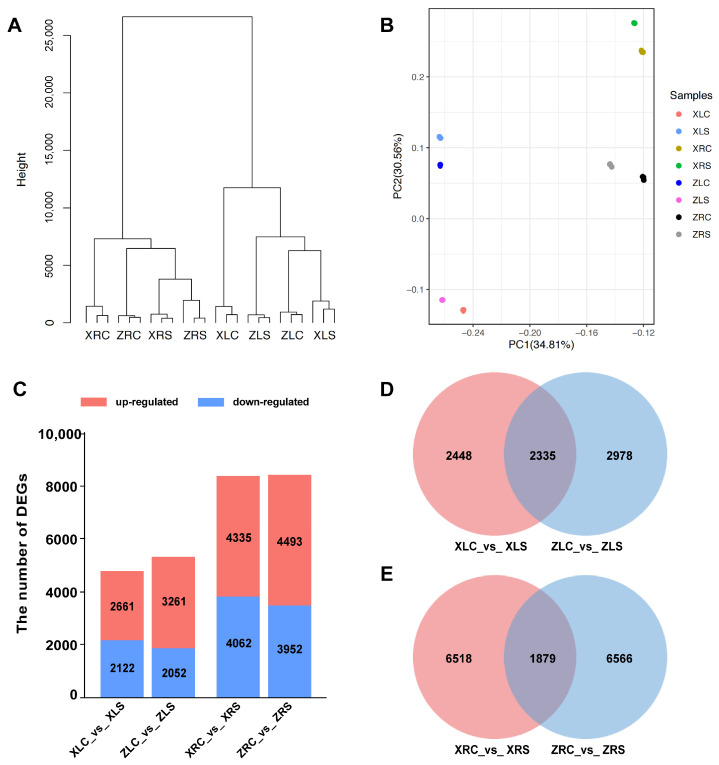
The relationship among 24 RNA-seq libraries and identification of the differential expression gene (DEGs). (**A**) Hierarchical clustering of RNA-seq samples. (**B**) PCA of RNA-seq samples. (**C**) The number of DEGs identified from four comparative groups. (**D**) Venn diagram of DEGs of leaves between X9 and Z9. (**E**) Venn diagram of DEGs of roots between X9 and Z9. RNA-seq libraries abbreviations: XLC: leaves of X9 cultivar under normal condition; XLS: leaves of X9 cultivar under salt stress; XRC: roots of X9 cultivar under normal condition; XRS: roots of X9 cultivar under salt stress; ZLC: leaves of Z9 cultivar under normal condition; ZLS: leaves of Z9 cultivar under salt stress; ZRC: roots of Z9 cultivar under normal condition; ZRS: roots of Z9 cultivar under salt stress.

We subsequently performed hierarchical clustering (Figure 3A) and a principal component analysis (PCA; Figure 3B) to estimate the similarity among the samples. As illustrated in Figure 3A, biological replicates were consistently grouped together. The samples from roots and leaves were clustered into two distinct groups. Within the root cluster, samples were organized according to the experimental conditions (salt stress and control samples). In contrast, the leaf cluster was differentiated primarily by cultivar, suggesting that leaves are less influenced by short-term salt treatment. A PCA further corroborated the strong reproducibility across biological samples, with each sample type forming a clearly defined group. Additionally, the two principal components effectively differentiated between the two cultivars and among various tissues of the same cultivar (Figure 3B). In summary, both hierarchical clustering and the PCA underscored the high sequencing quality and reproducibility of our samples.

### 2.3. Differential Expression Gene Analysis

To explore the molecular mechanisms underlying the response to salt stress in the two soybean cultivars, differential expression genes (DEGs) were identified from four comparison groups involving salt and control conditions, with each cultivar subjected to salt stress in both roots and leaves (Figure S1). A fold change ≥ 2 and FDR < 0.05 were used as standards for screening DEGs in response to salt stress. Compared with the control group (normal condition), 13,180 DEGs were identified in X9, including 6996 up-regulated genes, 6184 down-regulated genes. A total of 13,758 DEGs were identified in Z9, including 7754 up-regulated genes and 6004 down-regulated genes (Figure 3C). Although the salt-tolerant cultivar X9 and salt-sensitive cultivar Z9 showed similar numbers of DEGs under salt stress, the number of DEGs in roots was much higher than the number in leaves in both soybean cultivars (Figure 3C).

We conducted a comparative analysis of differentially expressed genes (DEGs) in the roots and leaves of two soybean cultivars, utilizing Venn diagrams for visualization (Figure 3D,E). Our findings revealed that there were 2335 shared DEGs in the roots and 1879 shared DEGs in the leaves between the two cultivars. Approximately 50% of the DEGs identified in the leaves were common to both cultivars, whereas only 30% of the DEGs in the roots were shared. The Venn diagrams further illustrated that the quantity of root-specific DEGs significantly exceeded that of leaf-specific DEGs in both the X9 and Z9cultivars (Figure 3D,E).

### 2.4. Transcriptome Data Verified by Quantitative Real-Time PCR

**Figure 4:**
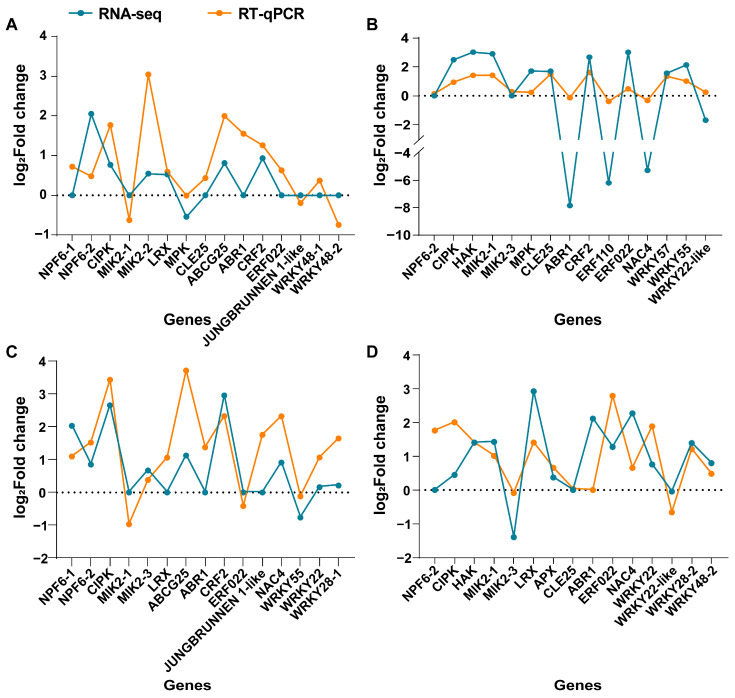
Comparison analyses of RT-qPCR and RNA-seq for 15 randomly selected DEGs in the leaves of X9 (**A**), the roots of X9 (**B**), the leaves of Z9 (**C**), and the roots of Z9 (**D**). The gene expression level of RNA-seq is shown as Log_2_(fold change), and the fold change is based on FPKM values of the salt stress group relative to the control group.

To verify the reliability of transcriptome sequencing data, we randomly selected 15 DEGs for quantitative real-time PCR (RT-qPCR) analysis (Table S2). The expression trends for both up-regulated and down-regulated genes were consistent between RNA-seq and RT-qPCR data for all 15 genes (Figure 4). In the roots of the X9 cultivar, the absolute values of gene expression levels measured by RT-qPCR were higher than those obtained from RNA-seq under salt stress conditions for genes such as ABR1, ERF110, and NAC4 (Figure 4B). These results corroborated the similar expression patterns of DEGs detected by RT-qPCR and RNA-seq.

### 2.5. Gene Ontology (GO) Enrichment Analysis for the DEGs

To explore the potential functions involved in DEGs, we performed a GO enrichment analysis including terms of biological process (BP), cellular component (CC), and molecular function (MF). In total, 5391 GO terms were identified as enriched in X9, with the DEGs in the leaves corresponding to 2189 GO terms (1286 BP, 266 CC, and 637 MF terms) and the DEGs in the roots corresponding to 3202 GO terms (1997 BP, 364 CC, and 841 MF terms) (Tables S3 and S4). For the Z9 sample, the DEGs were associated with a total of 6293 GO terms, of which 2473 GO terms (1465 BP, 298 CC, and 710 MF terms) were enriched in the leaves, while 3820 GO terms (2361 BP, 453 CC, and 1006 MF terms) were enriched in the roots (Tables S5 and S6). A total of 3587 GO terms were found to be common between X9 and Z9 (Figure S2), with 1328 (39.83%) of these terms shared in the leaves (758 BP, 178 CC, and 392 MF terms; Figure S2A) and 2259 (47.43%) shared in the roots (1418 BP, 262 CC, and 579 MF terms; Figure S2B).

**Figure 5:**
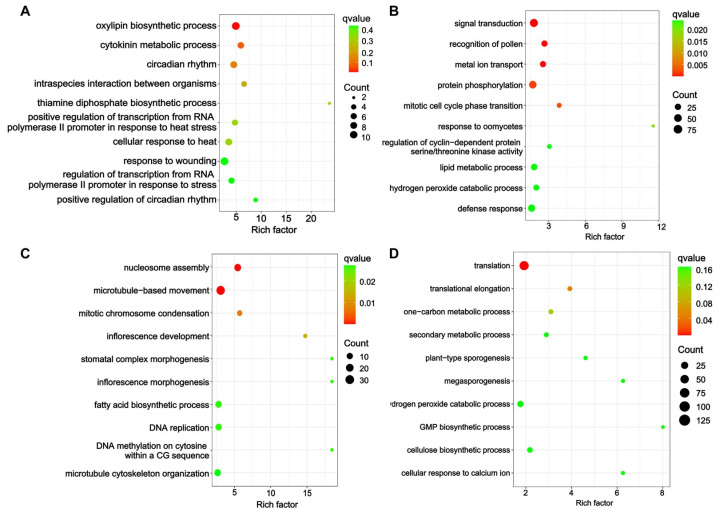
The top ten biological processes (BPs) identified through Gene Ontology (GO) enrichment in two soybean cultivars subjected to salt stress based on the different DEG sets. (**A**) Leaves of X9 cultivar. (**B**) Roots of X9 cultivar. (**C**) Leaves of Z9 cultivar. (**D**) Roots of Z9 cultivar. The color scale indicates *q* values from low values in red to high values in green. The size of the circle indicates the number of DEGs enriched in a particular GO term. Rich factor represents the degree of GO enrichment.

The GO enrichment analysis indicated differences between the two soybean cultivars. We compared the top ten GO terms for the BP, CC, and MF components between the two soybean cultivars based on a *q* value < 0.05, respectively (Figure 5, Figures S3 and S4). For BP, most of the DEGs of the X9 cultivar were enriched for metabolic processes in both leaves and roots, such as the oxylipin biosynthetic process, cytokinin metabolic process, and lipid metabolic process (Figure 5A,B). For CC and MF, the DEGs of X9 were also mostly enriched for enzymes and complexes associated with metabolic processes (Figures S3A,B and S4A,B). However, the DEGs of the Z9 cultivar were enriched for GO terms mostly associated with the cell cycle, such as nucleosome assembly, microtubule-based movement, translation, and structural constituent of ribosome (Figure 5C,D, Figures S3C,D and S4C,D). These results displayed the different responses to salt stress between the salt-tolerant X9 cultivar and salt-sensitive Z9 cultivar.

### 2.6. Kyoto Encyclopedia of Genes and Genomes (KEGG) Enrichment Analysis for the DEGs

**Figure 6:**
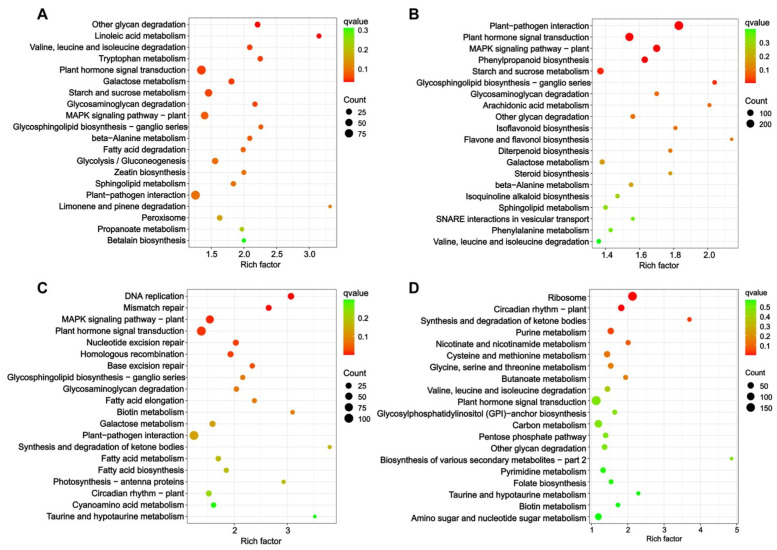
The top 20 biological processes identified through Kyoto Encyclopedia of Genes and Genomes (KEGG) enrichment in two soybean cultivars subjected to salt stress based on the different DEG sets. (**A**) Leaves of X9 cultivar. (**B**) Roots of X9 cultivar. (**C**) Leaves of Z9 cultivar. (**D**) Roots of Z9 cultivar. The color scale indicates *q* values from low values in green to high values in red. The size of the circle indicates the number of DEGs enriched in a particular KEGG pathway. Rich factor represents the degree of KEGG pathway enrichment.

**Figure 7:**
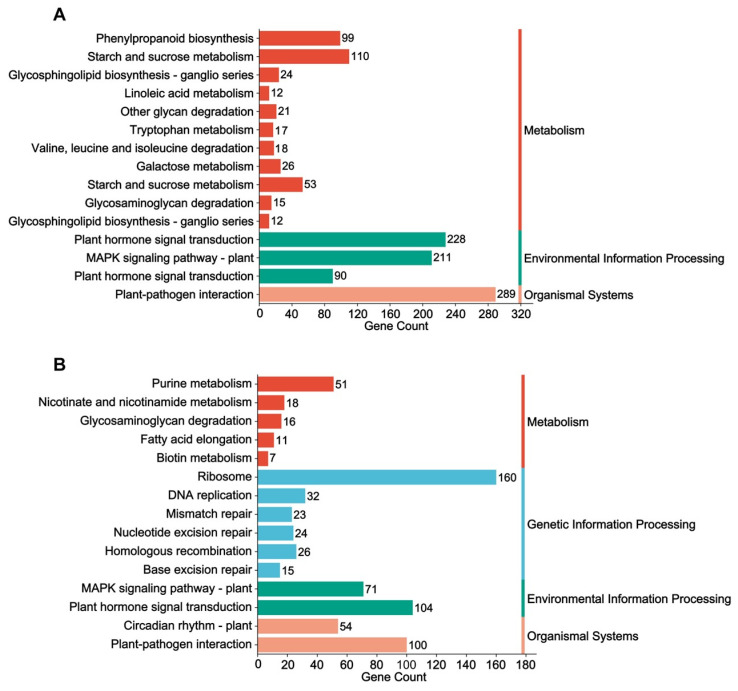
The significant enrichment of the KEGG pathway was categorized into four different groups in X9 (**A**) and Z9 (**B**). The values displayed to the right of the bars indicate the number of DEGs.

Consistent with the results of the GO enrichment, the KEGG enrichment analyses also showed a significant difference between the two soybean cultivars (Table S7). The top 20 significantly enriched pathways (*q* value < 0.05) in X9 were primarily associated with metabolic processes, including pathways related to linoleic acid metabolism, other glycan degradation, starch and sucrose metabolism, and phenylpropanoid biosynthesis (Figure 6A,B). In contrast, the DEGs in Z9 were significantly enriched in pathways related to the cell cycle, including the pathways for DNA replication, ribosome, circadian rhythm, mismatch repair, and nucleotide excision repair (Figure 6C,D). We categorized these significantly enriched pathways into four distinct groups, thereby elucidating the differences between the two soybean cultivars (Figure 7). Plant hormone signal transduction and MAPK (mitogen-activated protein kinase) signaling pathways were commonly enriched in both soybean cultivars. Among the metabolic pathways that were specific to the salt-tolerant X9 cultivar, starch and sucrose metabolism, as well as phenylpropanoid biosynthesis, exhibited the highest number of DEGs. Conversely, the ribosome pathway displayed the greatest number of DEGs in the Z9 cultivar. Furthermore, a significant distinction was observed in that the pathways associated with the cell cycle, which were enriched in Z9, were not significantly enriched in X9 (Figure 7).

### 2.7. Gene Co-Expression Modules Reveal Diverse Expression Patterns under Salt Stress

**Figure 8:**
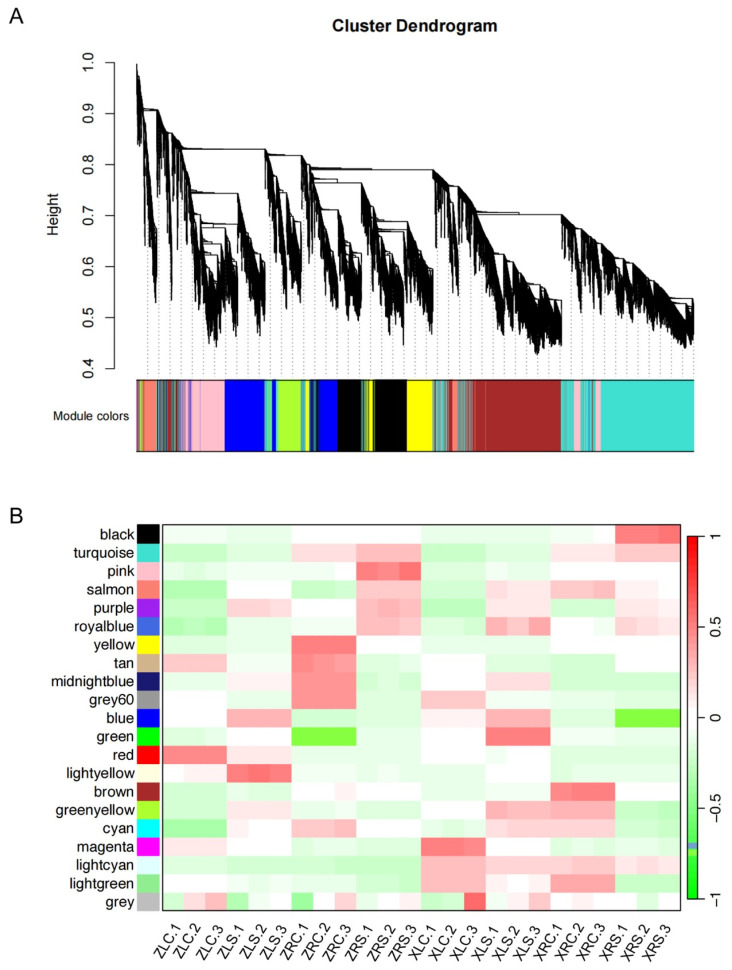
Construction of the co-expression gene modules. (**A**) Cluster dendrogram presents 21 co-expression modules labeled with different colors. (**B**) Correlation between samples’ expression patterns for 21 co-expression modules. The color scale indicates correlation coefficients with positive correlations in red and negative correlations in green between the modules and samples. White indicates there is no correlation.

The response of soybean to salt stress encompasses a variety of processes and the synergistic action of multiple genes. We here utilized a WGCNA to detect the gene co-expression networks associated with soybean response to salt stress. A total of 21 gene co-expressed modules labeled with different colors were determined based on FPKM values of 43,267 genes (Figure 8A). The sizes of these modules varied, ranging from 272 genes (royalblue) to 7816 genes (turquoise). Subsequently, we calculated the correlations between the module eigengenes and salt stress (Figure 8B) and examined the number of DEGs from four comparative groups distributed across these modules (Table 1). These modules exhibited a high overlap rate with the DEG sets in the roots for both soybean cultivars, such as the black, gery60, and royalblue modules in X9 and the pink, yellow, and midnightblue modules in Z9 (Table 1). In these modules, the black, royalblue, and pink modules showed significantly positive correlations with the samples of salt stress, while the grey60, yellow, and midnightblue modules showed significantly negative correlations with the samples of salt stress (Figure 8B; Kendall’s test, *p* < 0.01). For the leaves, only the salmon and lightgreen modules displayed more than a 30% overlap rate with the DEG set (Table 1).

**Figure 9:**
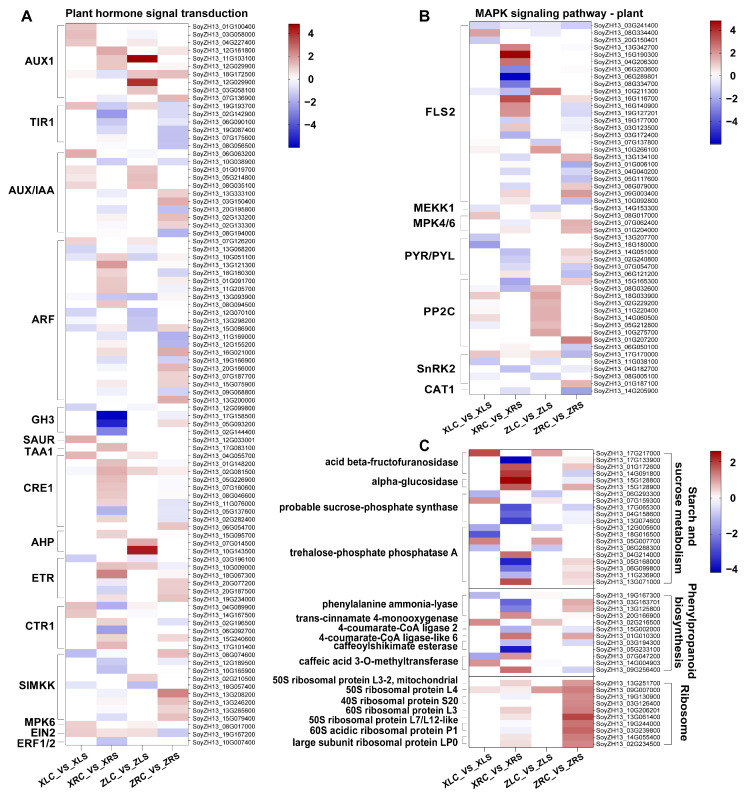
The changes in expression level of DEGs in four comparison groups for differential expression analyses involved in plant hormone signal transduction (**A**), MAPK signaling pathway (**B**), and three cultivar-specific pathways, including starch and source metabolism, phenylpropanoid metabolism, and ribosome pathways (**C**). The color scale indicates the values of log2(FoldChange). RNAseq library abbreviations: XLC: leaves of X9 cultivar under normal condition; XLS: leaves of X9 cultivar under salt stress; XRC: roots of X9 cultivar under normal condition; XRS: roots of X9 cultivar under salt stress; ZLC: leaves of Z9 cultivar under normal condition; ZLS: leaves of Z9 cultivar under salt stress; ZRC: roots of Z9 cultivar under normal condition; ZRS: roots of Z9 cultivar under salt stress.

We noticed that the purple module showed a positive correlation with samples subjected to salt stress and a negative correlation with normal samples across all four comparison groups (Figure 8B), indicating the purple module may contain the common regulatory pathway in X9 and Z9 in response to salt stress. We subsequently found that the purple module included many genes associated with the MAPK and plant hormone signal transduction pathways, which were significantly enriched in all four DEG sets (Figure 7), such as LRR receptor-like serine/threonine-protein kinase FLS2 and the serine/threonine-protein kinase SRK2A in the MAPK signaling pathway (Figure 9A), as well as auxin-responsive protein IAA11 (AUX/IAA11), mitogen-activated protein kinase kinase 4/5 (MAPKK4/5), and auxin response factor 19 (ARF19) in plant hormone signal transduction (Figure 9B).

In addition, several cultivar-specific pathways mentioned above were also found in modules significantly correlated with the samples of salt stress. The phenylpropanoid biosynthesis pathway was specifically enriched in the salt-tolerant X9 cultivar. A total of 99 DEGs associated with phenylpropanoid biosynthesis were up-regulated in the salt-tolerant X9 cultivar in response to salt stress. Notably, 68.69% of these DEGs exhibited, on average, a higher expression level in the salt-tolerant X9 cultivar under salt stress when compared to the salt-sensitive Z9 cultivar. This observation suggests that these DEGs may play a beneficial role in enhancing salt tolerance. Several DEGs encoding key enzymes in the phenylpropanoid biosynthesis pathway are found in the black and blue module and are positively and negatively correlated with salt stress, respectively (Figure 8B), such as phenylalanine ammonia-lyase, 4-coumarate-CoA ligase, and caffeoylshikimate esterase (Figure 9C). Moreover, we also found many DEGs in X9 encoded key enzymes in the starch and sucrose metabolism pathway in these two modules (Figure 9C), including acid beta-fructofuranosidase, alpha-glucosidase, etc. For the salt-sensitive Z9 cultivar, we found that the pink (which indicated a positive correlation with samples of salt stress) and yellow (which indicated a positive correlation with control samples) modules contain many DEGs associated with cell cycle pathways, especially those shown in Table 1 (Figure 8B). Ribosome biogenesis is a central process in the cell cycle. These DEGs encode a number of different ribosomal subunits and their expression was significantly induced in the salt-sensitive Z9 cultivar under salt stress (Figure 9C). In comparison, the expression of the same genes showed no significant changes in the salt-tolerant X9 cultivar under salt stress.

## 3. Discussion

Salt stress represents a considerable constraint on plant growth and development. Crosstalk between various signaling pathways often complicates the elucidation of underlying molecular mechanisms. In this study, we conducted a comparative analysis of the transcriptomes of two local soybean cultivars from Xinjiang, China. We performed GO term and KEGG enrichment analyses on four DEG sets. Subsequently, we clustered gene co-expression modules and concentrated on identifying pathways that exhibited both similarities and significant differences between the salt-tolerant X9 cultivar and the salt-sensitive Z9 cultivar.

### 3.1. Photosynthetic Response to Salt Stress by Soybean Seedlings

Soybean plants exposed to increasing durations of salt stress showed a gradual curling and yellowing of their leaves. The growth of the salt-sensitive Z9 cultivar was more severely inhibited than the salt-tolerant X9 cultivar. The ROS accumulation is a common secondary stress produced by plants in response to external environmental stresses, including salt stress [11,12]. Both NBT and DAB staining indicated that the accumulations of O_2_^−^ and H_2_O_2_ in the salt-sensitive Z9 cultivar are higher than the salt-tolerant X9 cultivar under salt stress. Furthermore, photosynthesis is a key metabolic process through which plants obtain energy. The cellular structure of plant leaves undergoes significant changes under salt stress, including impeded photosynthesis and a decreased photosynthetic rate [24,25,26]. Y(Ⅱ) was significantly higher in X9 leaves than in Z9 under salt stress, indicating that X9 is a significantly more salt-tolerant soybean cultivar than Z9 because the photosynthetic changes can effectively capture the salt tolerance characteristics of the plant [26,27]. Therefore, we confirmed that X9 is a more salt-tolerant soybean cultivar, while Z9 is a salt-sensitive soybean cultivar. In addition, changes in photosynthesis at different time points of salt stress in both cultivars also helped us to determine the time points of sampling for transcriptome sequencing.

### 3.2. Changes in Gene Transcription and Different Pathways of Soybean Seedings under Salt Stress

Although salt stress can be detrimental to soybean growth and development, soybean plants also exhibited positive physiological responses under salt stress. Here transcriptome analyses of two soybean cultivars were used to explore the salt-responsive metabolic pathway and DEG response to salt stress. Similar numbers of DEGs were detected in both the leaves and roots of the two soybean cultivars. However, the number of DEGs was higher in the roots than the leaves of both soybean cultivars (Figure 3). This result is consistent with that found for the salt-tolerant soybean line HJ-1 under salt stress, in which the number of DEGs in the roots was higher than that in the leaves under salt stress [28]. It has also been observed in other plants, as root tissue is the main organ that is in direct contact with high-salt environments [29,30,31]. In addition, approximately half of the DEGs in the leaves overlap between the two soybean cultivars, while this proportion is less than 30% in the roots, suggesting that there are more cultivar-specific DEGs responding to salt stress in the roots. Differences in response to salt stress between the two soybean cultivars may derive mainly from the initial root response to salt stress, while later responses to other aboveground organs may converge to be similar.

The GO enrichment analysis showed that the distribution of enriched DEGs under salt stress was different between the salt-tolerant X9 and the salt-sensitive Z9 cultivars. Fundamental metabolic processes are the main strategy of the salt-tolerant X9 cultivar in response to salt stress, and the DEGs of Z9 are mainly significantly enriched to cell cycle processes. This may reflect different strategies being used to respond to salt stress between the salt-tolerant and salt-sensitive soybean cultivars. This result was also observed in another study which indicated that the DEGs of the salt-tolerant cultivar ‘Qi Huang No. 34’ (QH34) were enriched for more metabolic processes while those of the salt-sensitive cultivar ‘Dong Nong No. 50’ (DN50) were enriched for ribosome biogenesis, a central process in the cell cycle [18].

### 3.3. Plant Hormone and MAPK Signaling Pathways Are Common Pathways Responding to Salt Stress in Soybean

We first found that the plant hormone signal transduction and MAPK signaling pathways exhibited responses to salt stress in both salt-tolerant and salt-sensitive soybean cultivars. This is consistent with previous studies on the soybean transcriptome in response to salt stress [18]. MAPKs are known to be activated by various biotic and abiotic stresses. Specifically, salt stress triggers the activation of two well-characterized MAPKs, which in turn, activate signaling molecules such as phosphatidic acid and ROS through the action of nicotinamide adenine dinucleotide phosphate (NADPH)-oxidase [32,33,34]. The most differentially expressed genes within the MAPK signaling pathway are some putative homologs of the receptor-like kinase flagellin-sensitive 2 (FLS2). FLS2 is a well-known immune signal receptor [35,36] that activates the downstream immune signaling pathways and induces the production of reactive oxygen species (ROS) [37,38,39]. Several studies have proposed that FLS2 induced by these biotic stresses also regulates plants’ responses to abiotic stresses, such as salt and drought stresses [40,41,42,43]. The DEGs in the type 2C protein phosphatase (PP2C) gene family in the MAPK signaling pathway were up-regulated in leaves of Z9 under salt stress, whereas they were almost unchanged in the other comparison groups (Figure 9B). It was shown that PP2C negatively regulates the MAPK signaling pathway in plants through the dephosphorylation of MAPK proteins [44,45,46]. In Arabidopsis, PP2C49 was highly expressed in root vascular tissues and its disruption enhanced plant tolerance to salt stress by regulating Na^+^ distribution under salt stress [46]. Not only that, PP2C also negatively regulates the plant hormone signal transduction that was significantly enriched by all four DEG sets (Figure 6). Plant hormones play a crucial role in regulating growth and development in plants, as well as in mediating responses to both abiotic and biotic stresses [16,17]. In the present study, we identified auxin as the most significantly enriched signal transduction pathway among plant hormones in both soybean cultivars. Auxin is instrumental in various aspects of plant growth, particularly in regulating root development and the proliferation of lateral roots. The expression levels of all twelve DEGs belonging to the auxin influx carrier (AUX1) gene family were significantly up-regulated under salt stress (Figure 9A). In addition, the expression levels of multiple DEGs classified as auxin/indole-3-acetic acid (Aux/IAA) and auxin response factor (ARF) gene families also responded to salt stress in both of the two soybean cultivars (Figure 9A). Aux/IAA family members have been identified as short-lived nuclear proteins that play a crucial role in repressing the expression levels of genes activated by ARFs [47,48]. Aux/IAA proteins have been suggested to bind with ARFs and prevent the activation of auxin-responsive genes in the absence of auxin [49,50,51,52,53]. This suggests the maintenance of plant growth when the auxin pathway is inhibited by salt stress.

### 3.4. The Cultivar-Specific Pathways Revealed the Different Responsive Strategies to Salt Stress between the Salt-Tolerant and Salt-Sensitive Cultivars

We subsequently obtained three cultivar-specific pathways as well. The pathways of starch and sucrose metabolism and phenylpropanoid biosynthesis were specifically enriched in the salt-tolerant X9 cultivar (Figure 6). Starch and sucrose are two of the most abundant carbohydrates in plants which play an important role in energy storage and supply. Starch and sucrose are crucial for responses to abiotic stress in plants, including salinity, drought, and low temperatures [54]. Sucrose can protect against damage caused by water stress as an osmolyte under drought and salt stress in plants [55]. Sucrose transport and distribution are also key processes for maintaining glucose homeostasis under abiotic stress [56]. We found that the sucrose and starch metabolic pathway had enrichment in 110 DEGs in the salt-tolerant X9 cultivar. Twenty DEGs encoding key enzymes in X9 exhibited significantly higher levels of gene expression changes under salt stress than Z9 (Figure 9C). Here, the alpha-glucosidase gene (SoyZH13_15G128800) was the most up-regulated gene in the salt-tolerant X9 cultivar. Hence, the X9 seedlings affected by salt stress are more likely to synthesize soluble sugar to alleviate osmotic stress and increase energy supply. Phenylpropanoids are a class of secondary metabolites in plants that are synthesized from phenylalanine and are essential for plant responses to salt stress [57]. In the present study, it was observed that 68.69% of DEGs associated with phenylpropanoid biosynthesis were up-regulated in response to salt stress. Furthermore, 81.72% of these DEGs showed on average higher expression levels in the salt-tolerant X9 cultivar when subjected to salt stress, in comparison to the salt-sensitive Z9 cultivar. This finding suggests that phenylpropanoids play a beneficial role in enhancing salt tolerance. The most up-regulated genes (SoyZH13_01G010300, SoyZH13_09G256400, and SoyZH13 20G166900) were annotated as encoding 4-coumarate-CoA ligase (4CL), caffeic acid 3-O-methyltransferase (COMT), and phenylalanine ammonia-lyase (PAL), respectively (Figure 9C). Consistent results were observed in a comparative transcriptome study based on the salt-tolerant soybean cultivar ‘QH34’ and the salt-sensitive soybean cultivar ‘DN50’ [18].

The ribosome pathway was significantly enriched in the salt-sensitive Z9 cultivar (Figure 6). Ribosome biogenesis and assembly are a central process in the process of the cell cycle. The alteration of any step in the ribosome biogenesis and assembly process may negatively impact cellular growth and induce proteotoxic stress [58]. Our comparison of the transcriptome of the salt-sensitive Z9 cultivar and the salt-tolerant X9 cultivar revealed that a majority of genes involved in the ribosome pathway are significantly up-regulated in the roots of Z9. Notably, the expression levels of several essential structural constituents of the ribosome, including differentially expressed genes (DEGs) encoding the 50S ribosomal protein L4 and the 50S ribosomal protein L7/L12-like, were found to be elevated (Figure 9C). In comparison, the same genes remained strongly down-regulated or slightly up-regulated in the root of the salt-tolerant X9 cultivar. The expressions of these DEGs encoding the structural constituent of the ribosome in the root of Z9 were higher than the X9. This result may reveal the salt-sensitive mechanism in the Z9 cultivar. Previous research has indicated that reduced ribosomal activity may play a critical role during the initial phases of cellular response to salt stress in soybean [18]. Our data showed the abundance of ribosomal proteins (Figure 9C) increasing significantly under low salt stress for the salt-sensitive Z9 cultivar, which suggests the extreme sensitivity of the Z9 cultivar to environmental salt concentrations results in the need for high ribosomal protein levels to maintain a normal cell cycle even under low-salt conditions, whereas the salt-tolerant X9cultivar, on the other hand, showed less variation to cope with low-salt conditions.

Our results proposed five important pathways responding to salt stress in soybean. Dissecting the cross-regulatory mechanisms between these pathways is key to breeding salt-tolerant soybean. In addition, some local salt-tolerant soybean cultivars are important genetic resource banks. As revealed in this study from the local cultivars, three cultivar-specific pathways (starch and sucrose metabolism, phenylpropanoid biosynthesis, and ribosome) provide important molecular modules for breeding salt-tolerant soybean.

## 4. Materials and Methods

### 4.1. Plant Materials and Experiment Design

The salt-tolerant soybean X9 cultivar and salt-sensitive soybean Z9 cultivar obtained from Xinjiang Academy of Agricultural and Reclamation Sciences were used in this study. Plump seeds were selected to be cultured in a laboratory kept at a constant temperature. We used the soil culture method (nutrient soil: vermiculite: perlite = 1:1:1) at 28/24 °C and under 16/8 h light/dark conditions for soybean culture experiments. Plants were watered every 3 days.

When the seedlings reached the stage with two real leaves and one heart leaf (~12 d), seedlings with similar growth vigor were selected and treated for 10 d with Hoagland nutrient solution containing 300 mM NaCl to observe the phenotypes of soybean seedlings. We then irrigated the soybean plants with a 300 mM NaCl solution, applying 200 mL for durations of 0 h, 1 h, 6 h, 12 h, and 24 h.

To clarify the time point of transcriptome sequencing after salt stress treatment, we analyzed the chlorophyll fluorescence of soybean leaves at each time point using an IMAGING-PAM chlorophyll fluorescence imager (Heinz Walz GmbH, Effeltrich, Germany). We selected the same area and the same shape (a circle with a diameter of 0.8 cm) of each leaf for detection. Three chlorophyll fluorescence parameters were derived directly from the instrument, including Fv/Fm, which reflected the estimation of the maximum photochemical efficiency of PSII, Y(II), which reflected the efficient quantum yield of photosystem II, and Y(NPQ), which reflected the yield of regulated energy dissipation of PSII. We compared the photosynthetic parameters at each time point of salt stress to determine the time point for transcriptome sequencing. Finally, the root tips and leaves were taken at 0 h, 1 h, 6 h, 12 h, and 24 h after salt treatment and placed in liquid nitrogen for freezing and then stored at −80 °C, respectively. Three biological replicates were performed for each sample in this study.

### 4.2. RNA Sequencing

Total RNA from each sample was extracted using RNA prep Pure Plant Kit according to the manufacturer’s instructions. NanoDrop 2000 (Thermo Fisher Scientific, Wilmington, DE, USA) was used to measure RNA concentration and purity. Agilent Bioanalyzer 2100 (Agilent Technologies, Santa Clara, CA, USA) and RNA Nano 6000 Assay Kit (Agilent Technologies, CA, USA) were used to assess RNA integrity. A total amount of 1 μg per sample was used for starting library construction. The cDNA sequencing libraries were constructed using a Hieff NGS Ultima Dual-mode mRNA Library Prep Kit for Illumina (Yeasen Biotechnology, Shanghai, China) following the manufacturer’s recommendations, and index codes were added to attribute sequences to each sample. All libraries were sequenced on an Illumina NovaSeq6000 platform (Illumina, San Diego, CA, USA) using 150 bp paired-end reads (PE 150).

### 4.3. Raw Sequencing Data Processing

Raw reads were processed using a cloud analytics platform (https://www.biocloud.net, BMKCloud, Beijing, China). First, adapters and reads with low quality were removed to obtain clean data. Q20, Q30, GC content, and sequence repeat levels were also calculated. All downstream analyses were based on high-quality clean data. Then, each read was aligned to the reference genome ‘Gmax_ZH13’ [59]. To eliminate the differences between samples, the gene expression level was normalized using the FPKM (Fragments Per Kilobase of exon model per Million mapped fragments) method.

### 4.4. Differential Expression Gene Analysis

Differential expression analysis of groups among the different conditions was performed using the DESeq2 1.4.4.0 R package [60]. The raw read counts were inputted to detect differentially expressed genes (DEGs). The absolute value of log2FoldChange ≥ 2 and a false discovery rate (FDR) adjusted to *p* ≤ 0.01 (according to Benjamini–Hochberg method) were classified as DEGs.

### 4.5. GO and KEGG Enrichment Analyses

The clusterProfiler 3.8.1 R package was used to perform GO enrichment analysis of DEGs [61]. Benjamini–Hochberg method was used to calibrate *p* value, and the significant GO terms were selected with *q* value below 0.05. KEGG enrichment was analyzed using KOBAS 2.0 software with the significance level set to *p* < 0.05 for enrichment [62].

### 4.6. Construction of Gene Co-Expression Modules

To identify gene co-expression networks, weighted gene correlation network analysis (WGCNA) was constructed using FPKM values to identify specific modules of co-expressed modules associated with salt stress [63]. We first checked for genes and samples with too many missing values using the *goodSamplesGenes* function in WGCNA 1.7.2 R package. We then removed the offending genes whose the last statement returns a ‘FALSE’ result. To construct an approximate scale-free network, a soft threshold power of five was used to calculate adjacency matrix for a signed co-expression network. Topological overlap matrix (TOM) and dynamic-cut tree algorithm were used to extract network modules. We used a minimum module size of 30 genes for the initial network construction and merged similar modules exhibiting > 75% similarity. To discover significantly drought-related modules, module eigengenes were used to calculate Pearson’s correlation with samples with different conditions.

### 4.7. qRT-PCR Analysis

To verify the reliability of RNA sequencing results, 15 DEGs were randomly selected from the transcriptome data for RT-qPCR. Based on the coding gene sequences, the RT-qPCR primers were designed using Primer Premier 6.0 software (Premier Biosoft Interpairs, Palo Alto, CA, USA), and Gmactin11 was selected as the internal control gene. The qPCR data were analyzed using the 2^−ΔΔCt^ quantitative method to determine differences in gene expression [64]. Three independent biological replicates and three technological replicates were used for each sample.

### 4.8. Data Processing and Visualization

In this study, we used SPSS 25.0 software to perform variance analysis and Duncan’s multiple comparison test. The bar diagram was plotted using Graphpad Prism 8.0. Venn diagrams, scatter diagrams, and heat maps were drawn using a cloud analytics platform (https://www.biocloud.net, BMKCloud, Beijing, China).

## 5. Conclusions

Our comparative transcriptome analysis between the salt-tolerant X9 cultivar and the salt-sensitive Z9 cultivar whose roots and leaves were placed under salt stress revealed the candidate genes’ and pathways’ response to salt stress. The phenotype and photosynthesis display an active response to salt stress in X9 but with rapid side effects relative to Z9. The number of DEGs in roots was much higher than in leaves in both of the soybean cultivars, and the number of root-specific DEGs was much higher than that of leaves, suggesting that roots are likely to be the priority organ to respond to short-term salt stress in soybean. A further GO/KEGG enrichment analysis suggested that the salt-tolerant X9 cultivar responded to salt stress by the processes of fundamental metabolism, and the salt-sensitive Z9 cultivar changed the processes of the cell cycle to respond to salt stress. We revealed that the common MAPK signaling pathway, plant hormone signal transduction, cultivar-specific starch and sucrose metabolism, phenylpropanoid biosynthesis, and ribosome biosynthesis may play crucial roles in soybean plants’ response to salt stress. The findings presented in this study enhance our comprehension of the molecular mechanisms associated with salt tolerance in soybean. The identified genes will serve as a valuable resource for the development of new soybean varieties exhibiting improved salt tolerance.

## Figures and Tables

**Table 1:** The number of DEGs that overlapped with the gene co-expression modules.

Modules	XL vs. XLS	XRC vs. XRS	ZLC vs. ZLS	ZRC vs. ZRS
black (2740) ^a^	125 (5%) ^b^	892 (33%)	226 (8%)	64 (2%)
turquoise (7815)	240 (3%)	354 (5%)	510 (7%)	296 (4%)
blue (4301)	179 (4%)	1235 (29%)	364 (8%)	263 (6%)
brown (4287)	328 (8%)	298 (7%)	212 (5%)	450 (10%)
yellow (3820)	175 (5%)	42 (1%)	157 (4%)	1326 (35%)
green (3586)	91 (3%)	1 (0%)	191 (5%)	727 (20%)
red (3413)	158 (5%)	1209 (35%)	151 (4%)	52 (2%)
pink (2505)	135 (5%)	63 (3%)	132 (5%)	984 (39%)
magenta (1682)	83 (5%)	106 (6%)	344 (20%)	113 (7%)
purple (1466)	66 (5%)	251 (17%)	47 (3%)	198 (14%)
greenyellow (979)	174 (18%)	91 (9%)	122 (12%)	89 (9%)
tan (822)	103 (13%)	54 (7%)	52 (6%)	66 (8%)
salmon (675)	224 (33%)	55 (8%)	17 (3%)	63 (9%)
midnightblue (467)	47 (10%)	11 (2%)	17 (4%)	173 (37%)
cyan (467)	16 (3%)	0 (0%)	17 (4%)	141 (30%)
lightcyan (331)	18 (5%)	15 (5%)	5 (2%)	23 (7%)
grey60 (312)	53 (17%)	105 (34%)	2 (1%)	2 (1%)
lightgreen (291)	4 (1%)	15 (5%)	91 (31%)	13 (4%)
lightyellow (282)	24 (9%)	7 (2%)	26 (9%)	36 (13%)
royalblue (271)	5 (2%)	87 (32%)	14 (5%)	2 (1%)

^a^ The values in parentheses represent the number of genes in the modules. ^b^ The percentages in parentheses represent the overlap rates between the modules and DEG sets.

## Data Availability

The raw sequencing RNA data are available at the Sequence Read Archive (SRA) under project accession PRJNA1147616. The data and materials that support the findings of this study are available from the corresponding authors upon reasonable request.

## Supplementary Materials

The following supporting information can be downloaded at: https://www.mdpi.com/article/10.3390/ijms25189818/s1.
