# Supplementary material for: Comparative Transcriptome Analysis of Salt-Tolerant and -Sensitive Soybean Cultivars under Salt Stress
Source: Int J Mol Sci. 2024 Sep 11;25(18):9818. doi: 10.3390/ijms25189818 (PMC11432363; doi:10.3390/ijms25189818)
Supplement: Supplementary file 1 [file ijms-25-09818-s001.zip › Supplementary_FiigureS1-S4.pdf]

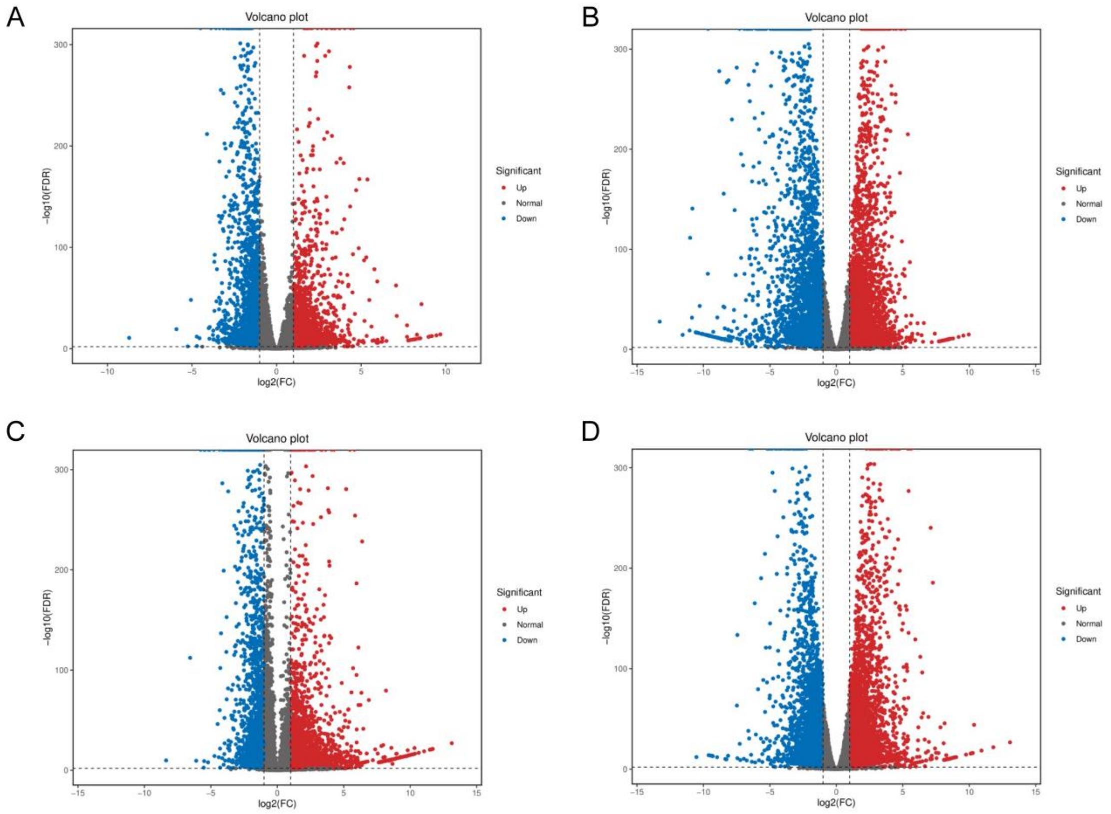

Supplementary Figure S1. The Volcano plots showed the results of differential expression gene analysis. (A) XLC vs XLS. (B) XRC vs XRS. (C) ZLC vs ZLS. (D) ZRC vs ZRS. RNA-seq libraries abbreviations, XLC: leaves of X9 cultivar under normal condition; XLS; leaves of X9 cultivar under salt stress; XRC: roots of X9 cultivar under normal condition; XRS: roots of X9 cultivar under salt stress; ZLC: leaves of Z9 cultivar under normal condition; ZLS: leaves of Z9 cultivar under salt stress; ZRC: roots of Z9 cultivar under normal condition; ZRS: roots of Z9 cultivar under salt stress.

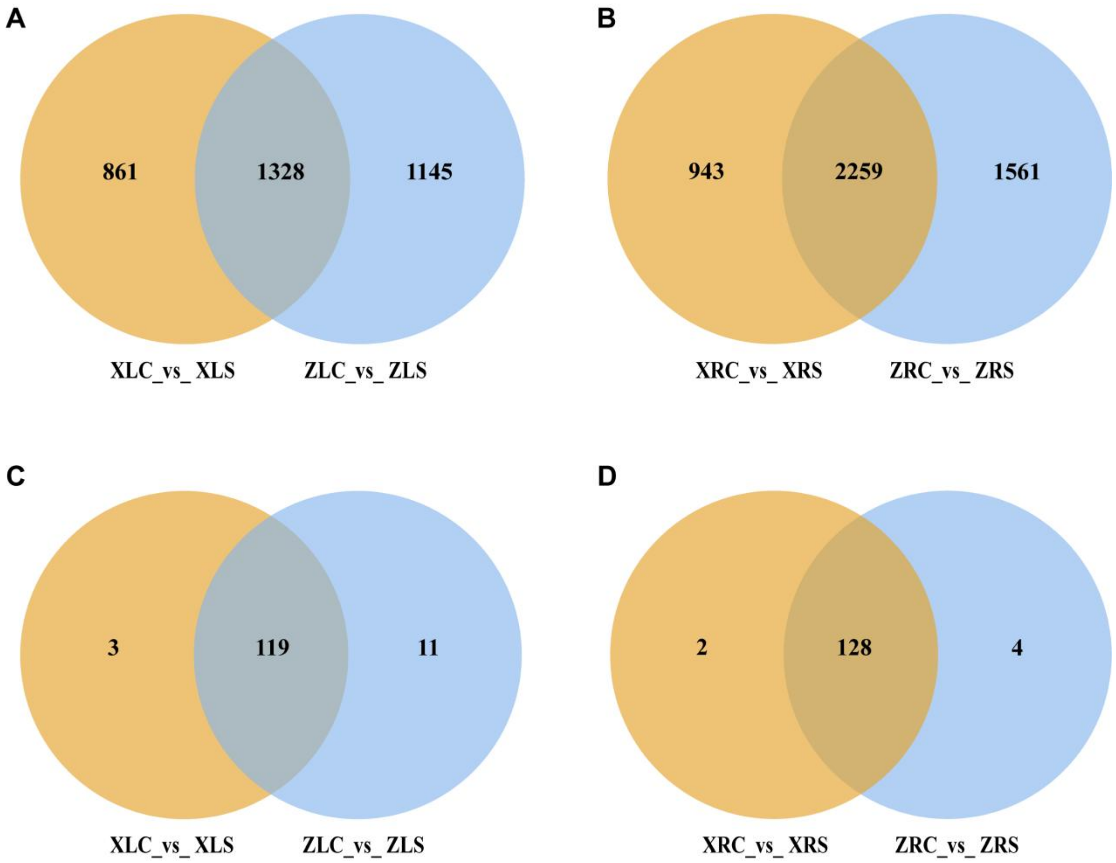

Supplementary Figure S2. The venn plots showed shared GO terms and KEGG pathways between two soybean cultivars. (A) Shared GO terms for leaf between X9 and Z9. (B) Shared GO terms for root between X9 and Z9. (C) Shared KEGG pathways for leaf between X9 and Z9. (D) Shared KEGG pathways for root between X9 and Z9. RNA-seq libraries abbreviations, XLC: leaves of X9 cultivar under normal condition; XLS; leaves of X9 cultivar under salt stress; XRC: roots of X9 cultivar under normal condition; XRS: roots of X9 cultivar under salt stress; ZLC: leaves of Z9 cultivar under normal condition; ZLS: leaves of Z9 cultivar under salt stress; ZRC: roots of Z9 cultivar under normal condition; ZRS: roots of Z9 cultivar under salt stress.

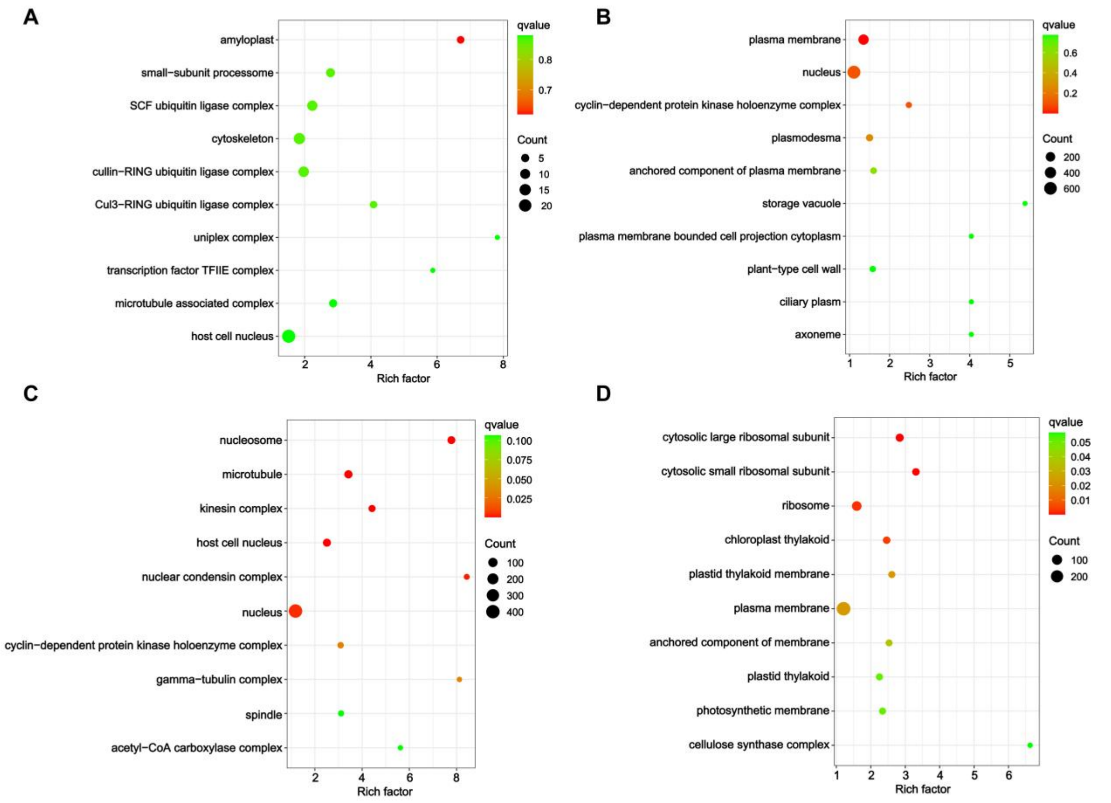

Figure S3. The top ten cellular components (CC) identified through Gene Ontology (GO) enrichment in two soybean cultivars subjected to salt stress based on the different DEG sets. (A) Leaves of X9 cultivar. (B) Roots of X9 cultivar. (C) Leaves of Z9 cultivar. (D) Roots of Z9 cultivar. The color scale indicates q values from low values in red to high values in green. The size of the circle indicates the number of DEGs enriched in a particular GO term. Rich factor represents the degree of GO enrichment.

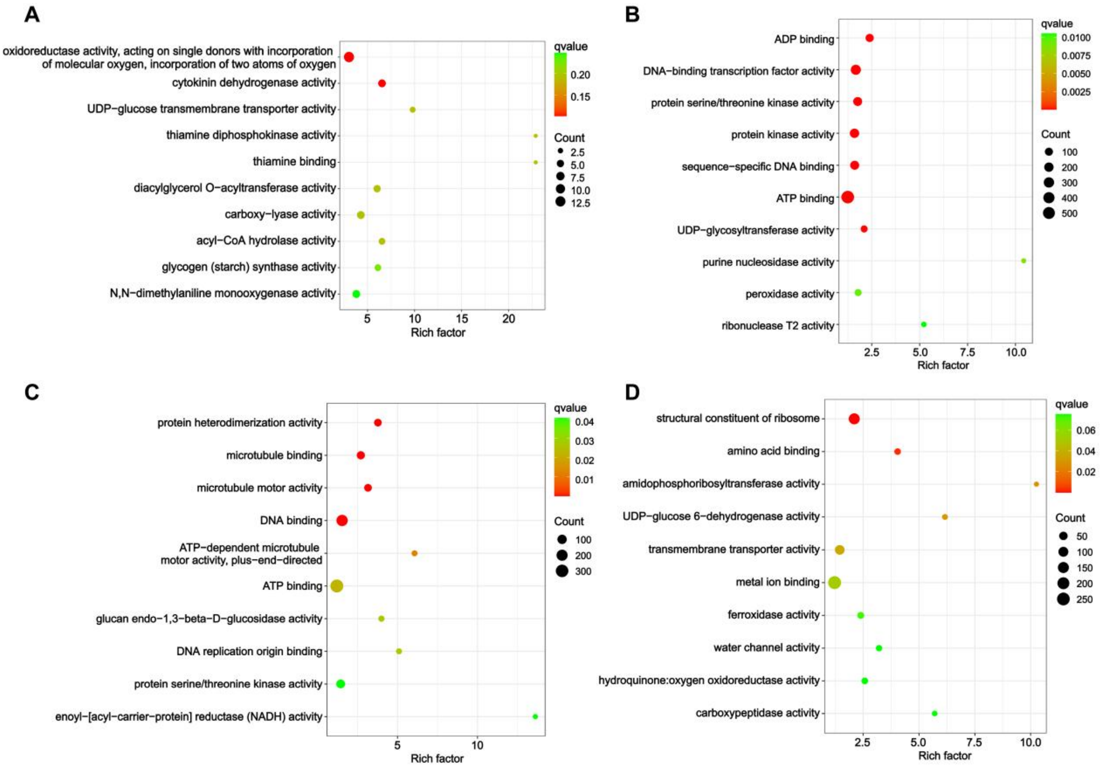

Figure S4. The top ten molecular functions (MF) identified through Gene Ontology (GO) enrichment in two soybean cultivars subjected to salt stress based on the different DEG sets. (A) Leaves of X9 cultivar. (B) Roots of X9 cultivar. (C) Leaves of Z9 cultivar. (D) Roots of Z9 cultivar. The color scale indicates q values from low values in red to high values in green. The size of the circle indicates the number of DEGs enriched in a particular GO term. Rich factor represents the degree of GO enrichment.
